# Optimal germination timing in unpredictable environments: the importance of dormancy for both among‐ and within‐season variation

**DOI:** 10.1111/ele.13461

**Published:** 2020-01-28

**Authors:** Hanna ten Brink, Jennifer R. Gremer, Hanna Kokko

**Affiliations:** ^1^ Department of Evolutionary Biology and Environmental Studies University of Zurich Winterthurerstrasse 190 CH‐8057 Zurich Switzerland; ^2^ Department of Evolution and Ecology University of California Davis CA 95616 USA; ^3^ Department of Evolutionary Biology and Environmental Studies University of Zurich Winterthurerstrasse 190 CH‐8057 Zurich Switzerland

**Keywords:** Bet hedging, competition, desert annuals, dormancy, environmental variation, evolution, germination phenology

## Abstract

For organisms living in unpredictable environments, timing important life‐history events is challenging. One way to deal with uncertainty is to spread the emergence of offspring across multiple years via dormancy. However, timing of emergence is not only important among years, but also within each growing season. Here, we study the evolutionary interactions between germination strategies that deal with among‐ and within‐season uncertainty. We use a modelling approach that considers among‐season dormancy and within‐season germination phenology of annual plants as potentially independent traits and study their separate and joint evolution in a variable environment. We find that higher among‐season dormancy selects for earlier germination within the growing season. Furthermore, our results indicate that more unpredictable natural environments can counter‐intuitively select for less risk‐spreading within the season. Furthermore, strong priority effects select for earlier within‐season germination phenology which in turn increases the need for bet hedging through among‐season dormancy.

## Graphical abstract

Here, we use a modelling approach to study the evolution of germination timing in annual plants living in unpredictable conditions. We find that when natural environments become more unpredictable, this counterintuitively leads to less within‐season bet hedging. Furthermore, in the presence of priority effects it is beneficial to spread germination over multiple years even when every growing season is favorable for reproduction.

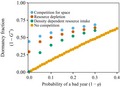

## Introduction

Most organisms live in variable and unpredictable environments, making it challenging for individuals to schedule important life‐history events such as emergence or reproduction. Seasonal precipitation, for example, can vary within‐ and among‐years, so poor timing can lead to drought mortality (Weekley *et al.* 2007). Bet hedging is defined as a strategy that reduces variance in fitness at a cost of a lower arithmetic mean fitness (Starrfelt & Kokko 2012), and it potentially allows organisms to deal with unpredictable conditions. The evolution of bet hedging is typically studied at a single time scale, for example either across or within growing seasons, yet variable conditions occur over multiple time scales (Gremer *et al.* 2016). Here, we study how risk‐spreading strategies that deal with unpredictability within a growing season interact with those dealing with across‐season variability.

Spreading germination of seeds (or hatching of eggs in case of animals) across years is an adaptation to stochastically varying growing seasons. Producing offspring with variable dormancy periods reduces the risk that all offspring germinate in a year with unfavourable conditions. Desert winter annuals are model organisms for this question, both empirically (Clauss & Venable 2000; Tielbörger *et al.* 2012; Gremer & Venable 2014; Gremer *et al.* 2016) and theoretically (Cohen 1966, 1967; Ellner 1985a, b). Bet hedging via dormancy has additionally been demonstrated in insects (Rajon *et al.* 2014; Grantham *et al.* 2016), rotifers (Tarazona *et al.* 2017), fish (Furness *et al.* 2015) and bacteria (Sturm & Dworkin 2015).

While spreading germination over multiple years can address variability among years, individuals also need to cope with unpredictability within a year. The start of a growing season, for example, varies across years. Individuals that germinate early in the season can profit from a long growing season, possibly yielding reproductive advantages (Ross & Harper 1972; Stratton 1992; Narita 1998; Donohue *et al.* 2010). Early emergence may also provide a competitive advantage for limited resources, if earlier establishment and growth provide a pre‐emptive advantage against later‐emerging competitors within a season (e.g. Verdú & Traveset 2005). However, mortality risks can be high early in the season (Mercer *et al.* 2011; Donohue 2014; Thomson *et al.* 2017), making early germination a ‘high‐risk high‐gain' strategy. Maternal plants may thus produce seeds that have different within‐year germination timing, possibly through differential provisioning of seeds (Simons & Johnston 2000) or heteromorphic seeds (Venable *et al.* 1995).

The variance reduction in bet hedging refers to fitness, not to phenotypes. Reduced fitness variance can be achieved by either large or small phenotypic variances, and the bet hedging literature assigns the labels ‘diversifying’ and ‘conservative’ bet hedging to these two options, respectively, although individuals can adopt a combination of these strategies (Haaland *et al.* 2019). Both are relevant when the phenotype is germination timing. Under within‐season unpredictability, individuals can produce offspring that germinate at different times within a season – a diversifying bet hedging strategy analogous to among‐year dormancy (Simons 2014; Poethke *et al.* 2016). Alternatively, individuals can adopt a conservative bet hedging strategy, by producing offspring that all germinate late. This foregoes any benefits of early growth (potentially leading to lower arithmetic mean fitness). On the other hand, such a strategy avoids the risk of all offspring dying early in the season should conditions be bad.

Despite several studies on the evolution of among‐season dormancy (Cohen 1966; Clauss & Venable 2000) and within‐season germination phenology (Metcalf *et al.* 2015; Poethke *et al.* 2016; Thomson *et al.* 2017), less is known about how these strategies interact. It is possible that germination of seeds at both time scales is due to variance in one trait only, a general responsiveness to germination cues, such that least responsive seeds germinate late within a season and some of this delay ‘spills over’ to create dormancy across years. Alternatively, timing may reflect variation in multiple traits, in which among‐year dormancy can be described as a ‘lock’ that first needs to open, with the responsiveness to cues thereafter determining how fast a seed germinates within a season (Finch‐Savage & Leubner‐Metzger 2006). Here we examine the evolution of within‐season phenology and among‐season dormancy (delaying germination for one of more years, hereafter referred to as dormancy) as separate traits. In doing so, we avoid assuming an *a priori* pattern of covariation, and we can ask what patterns of covariation would be favoured by natural selection. Keeping the traits distinct in our model is also informative regarding potential selection to reduce the correlation between dormancy and within‐season germination phenology. Furthermore, empirical data support describing these as separate traits, as evidenced by noisy and/or non‐significant covariation of within‐ vs. among‐season germination patterns in annual plants (Gremer *et al.* 2016; Torres‐Martínez *et al.* 2017).

Here, bet hedging theory creates some prior expectations. If an organism adopts one type of bet hedging strategy, variance in fitness is reduced, leaving less room for any further reduction of variance (Starrfelt & Kokko 2012). The presence of one risk reduction strategy therefore reduces the need for another. This expectation is, however, not always supported. For example, dispersal and dormancy both can act as bet hedging. Dispersal does so if it reduces mean success due to the costs of dispersal, but uncouples the fates of offspring (worst‐case scenarios of all occupying poor habitats simultaneously are avoided, Kokko & Starrfelt 1999). However, they are not wholly interchangeable (Venable & Brown 1988; Buoro & Carlson 2014) because the demographic consequences of each mechanism differ.

Here, we aim to understand the evolutionary interactions between within‐season germination phenology and dormancy. We use a modelling approach to study the separate evolution of these traits, as well as their joint evolution. Our model is inspired by annual plants living in an unpredictable environment where plants experience variable abiotic conditions, such as precipitation, and biotic interactions, such as intraspecific competition. We assume some years are suitable for survival and reproduction, others are not, and that early in the growing season harsh abiotic conditions can kill seedlings, for example via the return of the last frost (Shimono & Kudo 2003) or a long drought period after a germination triggering rain (Harrison *et al.* 2018). Early seedlings thus benefit from a longer growing season but risk early mortality (e.g. Purrington & Schmitt 1998). Late germination may be a safer strategy given abiotic conditions, yet with density dependence, individual fitness not only depends on its own germination and the environmental conditions it encounters, but also on the phenology of others (Pantastico‐Caldas & Venable 1993; Gremer & Venable 2014; Metcalf *et al.* 2015; Leverett & Shaw 2019). For example, early individuals may pre‐emptively take up resources, decreasing resource availability for latecomers (priority effects). Therefore, late germination may reduce abiotic risk at the expense of succeeding in competition. Thus, we expect intraspecific competition to affect the evolution of germination strategies in variable environments.

In the present work, we show that dormancy compensates for within‐season bet hedging from germination phenology. Spreading germination across years reduces the risk that all members of an early germinating lineage encounter bad early conditions. Because of this, when the probability of encountering a bad year is high, individuals produce seeds that are highly dormant, and when they germinate they do so early within the season. Furthermore, in the presence of priority effects (where early germinating individuals reduce resources or available space), dormancy is selected for even when every growing season is favourable for reproduction. This occurs because priority effects select for early within‐season germination, but that risky strategy is only favoured if germination is also spread across multiple years.

## Models and analysis

We use five models to examine the interaction between dormancy and within‐season germination phenology (for detailed model descriptions see Appendix S1). We follow Finch‐Savage & Leubner‐Metzger (2006) and define dormancy as a block to the completion of germination of viable seeds under favourable conditions, mathematically expressed by the dormancy fraction (proportion of seeds that stay dormant from one year to the next). Within‐season germination phenology determines the time it takes a seed to germinate after it has broken free from its dormant state. While it is possible to treat within‐season germination phenology and dormancy as a single trait, we treat them as two independent traits in order to understand how they interact in the absence of any constraints.

We focus on a population of annual plants inhabiting a seasonal environment, where each year consists of a growing season and a non‐growing season. The quality of the growing seasons varies among years, and for simplicity we assume years to be either suitable (‘good’) or completely unsuitable (‘bad’). Reproduction is impossible in bad years, and given that we model annuals, dormant seeds are the only way for a lineage to survive such years. Within good years, we assume uncertainty in abiotic conditions early in the growing season – in some years mortality is high and growth conditions are poor early in the season and improve with time; in other years conditions are already favourable early in the season. Seeds can neither predict the start of continuously favourable growing conditions, nor the quality of a year (e.g. an early rain might be followed by a prolonged period without precipitation). We consider seeds responding to environmental cues only implicitly; fast responders (e.g. germinate after the first rain) are those whose timing within a season is always earlier than that of slow responders (e.g. requiring many rain events). Our models simplify precise weather patterns, while retaining the effect, that is the phenological outcome, which we assume to be the trait under selection.

Our *Analytical model* extends the classic model of Cohen (1966), and assumes three types of years: bad years (probability 1-φ) that are not suitable at all, late years (probability $\varphi \left(1-\rho \right)$) suitable for late‐germinating seeds and early years (probability φρ), suitable for all germinating seeds. The phenology of seeds follows a similar structure. A seed breaks from its dormant state with probability G, and if it does, it germinates early with probability $p_{e}$, and late with probability $1-p_{e}$. Early seedlings yield plants with per capita seed production of $Y_{e}$ in early years. In late years, early germination is lethal, while late‐germinating seeds yield plants with per capita seed production of $Y_{\ell },$ irrespective of the conditions early in the season. Since early years reward early seedlings with more time to grow and improve their per capita yield, we assume that $Y_{e}=c_{e}Y_{\ell }$, where $c_{e}>1$ is the relative benefit of germinating early. Individuals do not directly compete with each other; the fitness of an individual depends only on its own strategy and on the abiotic conditions it encounters. While this model is an oversimplification of reality, obtaining analytical solutions in the absence of numerous additional effects forms a useful baseline against which to compare more complicated models, below.

With the *Continuous season model*, we relax the assumption of discrete categories of years, and assume continuous variation in the onset of favourable growing conditions (the day from which on favourable growing conditions prevail for the rest of the season). To do so, we extend the model of Poethke *et al. *(2016) and allow for the evolution of both the mean and variance of within‐season emerging timing. Here, a conservative bet hedging strategy is characterised by a late mean within‐season germination date, $\bar{E}$, which reduces the risk of emerging before the season has switched to offering reliably favourable growing conditions until the season end. An alternative response is to adopt a diversified bet hedging strategy by evolving a high variance parameter ϵ, such that some offspring of an individual germinate early in the season, others late. An increase of both the mean and variance in within‐season emergence date combines the aspects of conservative and diversified bet hedging.

To study how competition affects the evolution of germination strategies, we investigate four submodels of the continuous season model (Table A1). For each day τ within the season of continuously favourable conditions, in good years all currently active individuals collect resources at a daily rate of $c\left(\tau \right)$. Since resource collection is impossible under bad abiotic conditions, some years offer more time to gather resources than others. In the *density‐independent model*, daily resource intake, $c\left(\tau \right)=c$, is independent of other individuals, reflecting, for example a situation where plants are sparsely distributed across the landscape. In the *resource‐depletion model*, a total amount *R* of resources is available at the beginning of the season. Individuals gather resources at a constant rate of $c\left(\tau \right)=c$ until the resource is depleted, after which daily resource intake equals $c\left(\tau \right)=0$. This scenario describes, for example, a situation where there is one rain event, after which water in the soil is depleted by the plants. In the *density‐dependent resource intake model*, daily resource intake decreases with the number of active individuals. In this scenario there is resource competition, where resource uptake in hindered by conspecifics. In the *competition‐for‐space model*, individuals compete for space, with early germinating individuals occupying space that is no longer available for latecomers. Due to each individual occupying its own site, resource intake is independent of other individuals.

For each model, we evaluated optimal germination strategies (dormancy, within‐season phenology or both) that maximise geometric mean fitness. The analytical model allows the optimal strategy to be calculated in closed form, the density‐independent continuous season model can be solved numerically. For the remaining three models we use individual‐based simulations (Appendix S2). For the models that allow solving for the optimal strategy either analytically or numerically, we also calculate the phenotype with the highest expected arithmetic mean fitness. Bet hedging is defined as a strategy that sacrifices some of the arithmetic mean fitness to achieve a reduction in variance of fitness, thus identifying the properties of an arithmetic mean maximiser allows us to check that the evolved strategies formally meet the criteria for bet hedging.

## Results

### Dormancy substitutes for within‐season bet hedging

First, we study how dormancy affects the evolution of within‐season germination phenology by assuming that bad years do not occur (φ=1), and individuals experience only uncertainty with respect to the start of favourable growing conditions. Under these assumptions, within‐season variation is the only reason for bet hedging to evolve, enabling clear interpretation of results.

In the analytical model, a sufficiently high relative benefit of emerging early, $c_{e}$, that allows the probability of an early year (ρ) to satisfy ρ $>1/c_{e}$, leads to a pure strategy of early seed germination ($p_{e}=1$) having the highest arithmetic mean fitness. However, in the absence of dormancy such a strategy cannot persist in the long‐term whenever late years exist. By producing some late seeds, lineage extinction in a late year is avoided. Such a strategy decreases both variance in fitness and mean arithmetic fitness and is therefore a bet hedging strategy.

**Figure 1:**
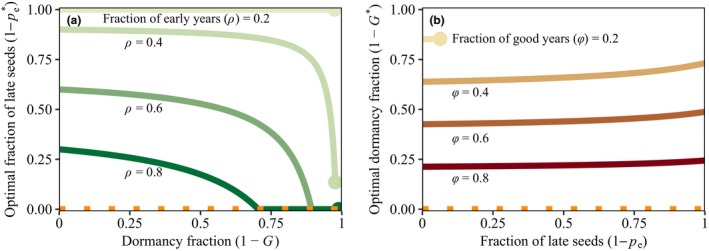
Optimal fraction of late seeds (a) and dormancy fraction (b) as a function of the other germination trait in the analytical model. When only a single trait evolves, dormancy can compensate for within‐season bet hedging (a), but not the other way around (b). (a), the optimal fraction of late seeds, $1-p_{e}^{*}$, as a function of the fraction of dormant seeds (1-G), that maximises geometric mean fitness. With higher dormancy, optimal fractions of late seeds are lower. (b), the optimal dormancy fraction, $1-G^{*}$, as a function of the fraction of late‐germinating seeds, that maximises geometric mean fitness. Higher fractions of late seeds lowers yield and therefore increases the need for dormancy. The horizontal orange‐dashed lines indicate the optimal strategy when maximising arithmetic mean fitness. In panel (a) the fraction of good years, φ, equals 1 and the fraction of early years, ρ, varies between 0.2 and 0.8, with darker colours indicating higher values. Note that when the fraction of early years is low (for values of $\rho <1/c_{e}$; lightest green line), individuals produce only late seeds and dormancy no longer compensates for within‐season bet hedging. In panel (b) the fraction of early years, ρ, equals 1 and the fraction of good years φ, varies between 0.2 and 0.8, with darker colours indicating higher values. The dots in (a) and (b) indicate a threshold value above which the population cannot persist. Other parameters are s=0.9, $Y_{\ell }=5$ and $c_{e}=3$.

If dormancy substitutes for within‐season bet hedging, the fraction of early germinating seeds will increase with dormancy. To see if this is true, we fix the annual germination fraction G and find how its value impacts the $p_{e}$ maximising long‐term growth rate (given in eqn A4). Since $p_{e}^{*}$ is a decreasing function of G, a situation with high dormancy (low G) implies more risk taking (high $p_{e}^{*}$) within a season (Fig. 1a). Dormancy therefore substitutes for within‐season bet hedging.

(1) $$p_{e}^{*}=\frac{c_{e}\rho -1}{c_{e}-1}\cdot \frac{\left(Y_{\ell }-s\right)G+s}{GY_{\ell }}.$$

In case $\rho <1/c_{e}$, both arithmetic and geometric mean fitness are maximised when individuals produce only late seeds ($p_{e}=0$, lightest green line in Fig. 1a). In this special case, dormancy patterns do not influence within‐season phenology.

The result that dormancy can substitute for within‐season bet hedging makes intuitive sense. A lineage that produces offspring that always germinate early, but spreads germination across years, will survive in the long‐term, even when some years only offer growth conditions late in the season. Late years become equivalent with unsuitable years if all seeds germinate early, and dormancy is sufficient to compensate for this.

**Figure 2:**
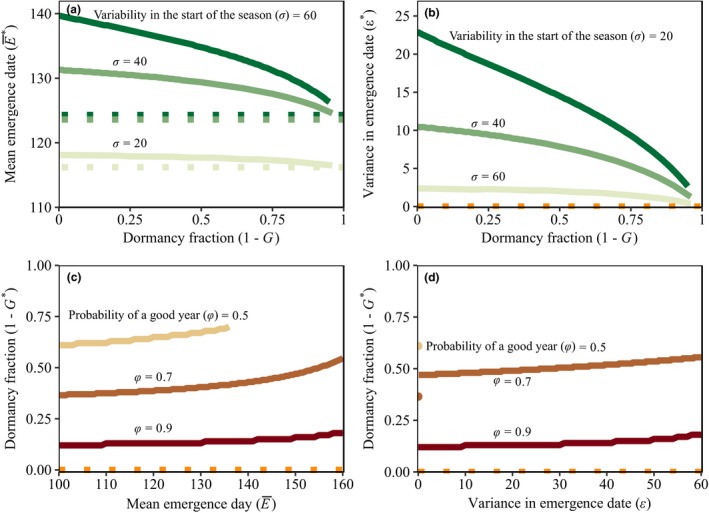
Optimal dormancy and within‐season phenology from the density‐independent continuous season model. Dormancy can compensate for within‐season bet hedging (panels a and b), but within‐season bet hedging results in more, instead of less, dormancy (panels c and d). This is true for conservative (panels a and c) and diversified (panels b and d) within‐season bet hedging. In panels (a) and (b) dormancy is fixed and both the mean emergence date, $\bar{E}$, and the variance in emergence, ε, can evolve. In panels (c) and (d) dormancy can evolve, while the parameters determining within‐season germination phenology are fixed. The horizontal‐dotted lines indicate the optimal strategy when maximising arithmetic mean fitness, solid lines indicate optimal strategies that maximise geometric mean fitness. In (a) and (b) the fraction of good years, φ, equals 1. The variability in the start of the season, σ, equals 20 (lightest green), 40 (green) or 60 (darkest green). In (c) and (d) there is no variation in the start of the season (σ=0) and the fraction of good years, φ, equals 0.5 (lightest brown), 0.7 (brown) or 0.9 (darkest brown). Note that for φ = 0.5 there is only a viable population in case there is no variation in the mean emergence date (ϵ=0). The variance in emergence, ϵ , equals 0 in panel (c). The mean emergence date, $\bar{E}$, equals 100 in panel (d). Other parameters are c=0.05, s = 0.9, ${\bar{S}}_{B}=100\;\;\text{and}\;\;{\bar{S}}_{E}=200$.

Because of the generality of the above reasoning, it is not surprising that the result extends to the density‐independent continuous season model (Fig. 2). In the absence of dormancy and with all years offering suitable growth conditions, the best strategy combines aspects of conservative and diversified bet hedging. The optimal mean germination date, ${\bar{E}}^{*}$, occurs late in the season (Fig. 2a), accompanied by high variance in germination date, $\epsilon^{*}$ (Fig. 2b). Both traits decrease with an increase in dormancy. Decreasing the fraction of good years decreases the range of dormancy fractions for which the population is viable, without affecting optimal within‐season germination timing.

### Within‐season bet hedging cannot substitute for dormancy

To investigate how within‐season germination phenology affects the evolution of dormancy, we assume no variation in the start of favourable growing conditions (ρ=1 in the analytical model, σ=0 in the continuous season model), but some years may be wholly unsuitable (φ < 1). Among‐season variation is therefore the only reason for dormancy to evolve. Relaxing this simplifying assumption does not affect the results (Appendix S3).

In the analytical model, we expect a decrease in the optimal dormancy fraction as a function of the fraction of late seeds, if late seeds substitute for dormancy. However, in case ρ=1 we find that the optimal germination fraction equals.

(2) $$G^{*}=\frac{\varphi Y_{\ell }\left(1+p_{e}\left(c_{e}-1\right)\right)-s}{Y_{\ell }\left(1+p_{e}\left(c_{e}-1\right)\right)-s},$$

which is an increasing function of $p_{e}$ as long as the relative benefit of emerging early is within the plausible range of $c_{e}>1$. Hence, as the fraction of late‐germinating seeds increases, more, instead of less, dormancy will evolve (Fig. 1b). Within‐season bet hedging therefore does not substitute for dormancy.

For an individual germinating in a bad year, within‐season timing of germination does not matter since reproductive success is zero. It is therefore not surprising that within‐season bet hedging does not substitute for dormancy. The increase in dormancy with an increase of the fraction of late seeds may appear more surprising, but this is due to lower yield of late seeds selecting for more dormancy.

We find similar results in the density‐independent continuous season model. As in the analytical model, the optimal dormancy fraction increases with more within‐season bet hedging, either via conservative (Fig. 2c) or diversified (Fig. 2d) bet hedging. As soon as the variance parameter ϵ>1 , there is a jump in the optimal dormancy fraction. This occurs because, on average, half the offspring of an individual germinate before the onset of favourable growing conditions. Their failure to reproduce results in a low yield, in turn selecting for more dormancy.

### Many good years result in high within‐season bet hedging

To understand the evolutionary interaction between dormancy and within‐season germination timing, we now turn to the joint evolution of these traits, assuming that individuals have to deal with both among‐ and within‐season variation.

**Figure 3:**
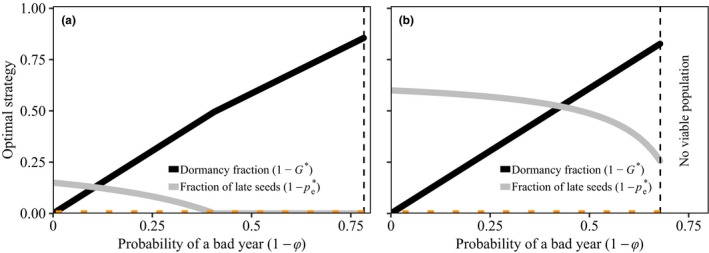
Optimal strategies as a function of the probability of encountering a bad year (1-φ) in the analytical model. As the chance of encountering bad years increases, the optimal fraction of early seeds increases. The horizontal orange‐dotted lines indicate the optimal strategies when maximising arithmetic mean fitness (G=1 and $p_{e}=1$). In panel (a) the fraction of early years,ρ, equals 0.9, in panel (b) the fraction of early years equals 0.6. Other parameters are s=0.9, $Y_{\ell }=5$ and $c_{e}=3$.

The optimal germination behaviour in the analytical model equals. The optimal between‐year germination fraction, $G^{*}$, is independent of the fraction of early seeds and the fraction of early years as long as $p_{e}=p_{e}^{*}$. The optimal fraction of early seeds, $p_{e}^{*}$, however, decreases with parameter φ, the probability of encountering a good year (Fig. 3). When most years are good, there is little dormancy (eqn 3a), and within‐season variation makes late germination of some seeds necessary. In contrast, when bad years are frequent, high dormancy fractions evolve, and thus, the benefit of late seeds decreases.

(3a) $$G^{*}=\frac{\varphi Y_{\ell }-s}{Y_{\ell }-s},$$

(3b) $$p_{e}^{*}=\frac{c_{e}\rho -1}{c_{e}-1}\cdot \frac{\varphi \left(Y_{\ell }-s\right)}{\varphi Y_{\ell }-s}.$$

**Figure 4:**
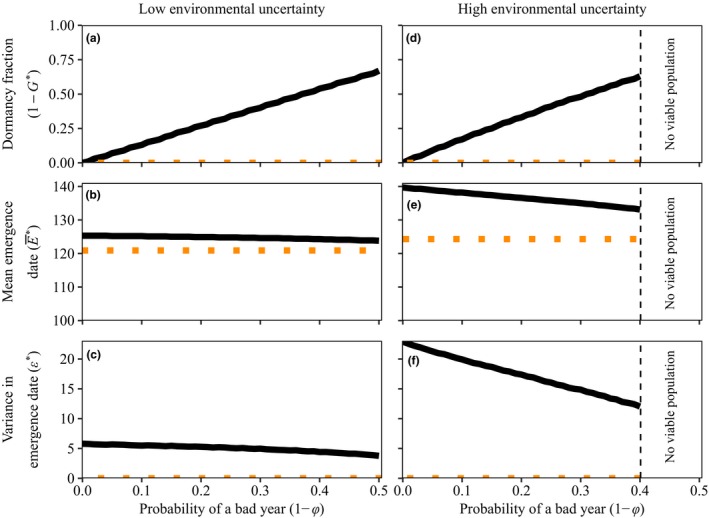
Optimal trait values as a function of the probability of a bad year (1-φ) for the density‐independent continuous season model. As the probability of encountering a bad year increases, the need for dormancy, $1-G^{*},$ also increases (panels a and d). Therefore, both the mean emergence date $\bar{E^{*}}$ (panels b and e) and the variance in emergence date $\epsilon^{*}$ (panels c and f) decrease. This effect is especially strong in case of high uncertainty about the start of favorable growing conditions (right column). The horizontal‐dotted lines indicate the optimal strategy when maximising arithmetic mean fitness. Parameters are c=0.05, s = 0.9, ${\bar{S}}_{B}=100\;\;\text{and}\;\;{\bar{S}}_{E}=200$. Parameter σ=30 in panels a, b and c, and σ=60 in panels d, e and f.

We find a similar result in the density‐independent continuous season model; with higher probability of a good year, less dormancy evolves, and later mean emergence day and more variance in emergence timing evolve (Fig. 4). The result is especially pronounced when σ, the maximum deviation of the start of favourable growing conditions, is high.

### Competition leads to earlier emergence

**Figure 5:**
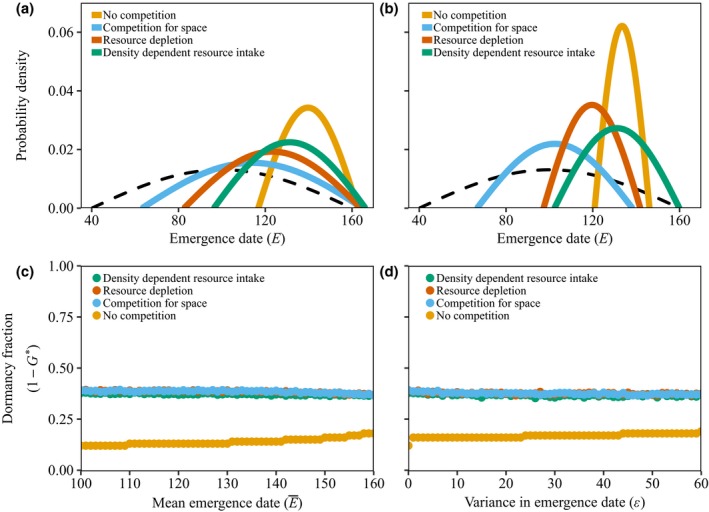
Emergence date E and dormancy fractions for the four different submodels of the continuous season model. The upper panels show the optimal distribution of emergence dates when there is no dormancy (panel a) or high dormancy (panel b, the dormancy fraction 1-G =0.6). The dashed black line corresponds to the distribution of the start of the favourable growing conditions (σ=60). All years are good and the fraction of dormant seeds is fixed in panels a and b. The lower panels show the optimal dormancy fraction as a function of the mean emergence date (panel c) or the variance in emergence (panel d). In these two panels, there is no variation in the start of favourable growing conditions (σ=0), and there is a probability of 0.1 to encounter a bad year where reproduction is not possible. Other parameters are as shown in Table B1

In all three competition models, fixed dormancy fractions create selection for an earlier mean emergence date and higher variance in emergence time compared to the density‐independent model (Fig. 5a and b). In the absence of direct competition, germinating late is a safe, conservative bet hedging strategy. However, with locally acting density dependence, individuals that germinate later than their competitors might not be able to reproduce at all. A strategy with a high variance in emergence date, ϵ , is now beneficial since it results in successful offspring in both early and late years.

As before, dormancy reduces the need for within‐season bet hedging. Increasing the fraction of dormant seeds decreases both conservative bet hedging, via a decrease in the optimal mean emergence date, ${\bar{E}}^{*}$, and diversifying bet hedging, via a decrease in the optimal variance in emergence, $\varepsilon^{*}$. This leads to more risk‐prone strategies, with no reproduction in extremely late years (Fig. 5b); the lineage can still survive due to dormant seeds.

In the absence of within‐season variation (σ=0 and ${\bar{S}}_{B}=100$), dormancy is substantially higher in models that include local competition (Fig. 5c and d). Competition reduces the number of offspring an individual produces, which leads to a higher fraction of dormant seeds.

**Figure 6:**
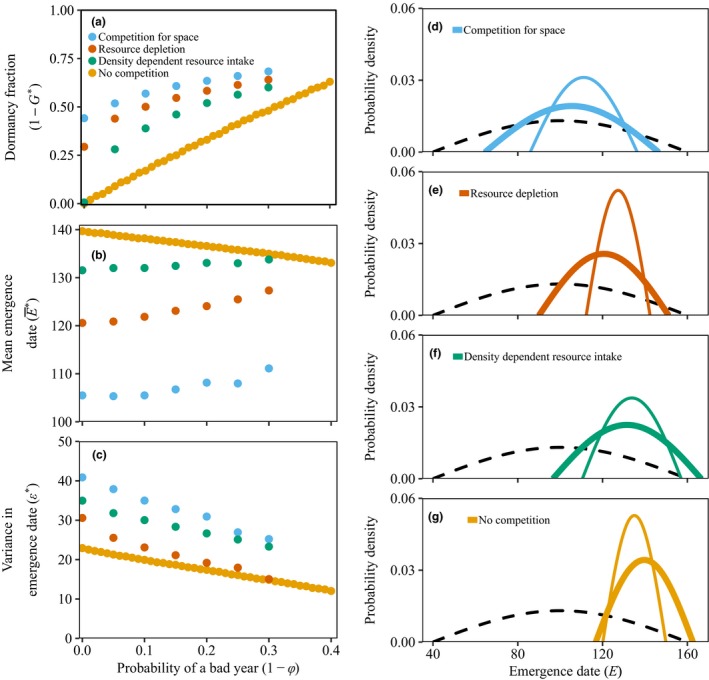
Optimal germination strategies in relation to good and bad years. The optimal fraction of dormant seeds $1-G^{*}$ (a), mean emergence date $\bar{E^{*}}$ (b) and variance in emergence date $\epsilon^{*}$ (c) as a function of the probability of encountering a bad year (1-φ) for the four continuous season models. The panels on the right (d‐g) show the optimal distribution of emergence dates when all years are good (thick line) and when 30% of the years are bad (thin line). The dashed black line corresponds to the distribution of the start of the favourable growing conditions (*σ* = 60). Other parameters are as shown in Table B1

Dormancy increases with the probability of a bad year in all models (Fig. 6a). In contrast to the density‐independent model, the mean emergence date increases slightly with the probability of a bad year (Fig. 6b) when all traits coevolve. However, since variance parameter $\epsilon^{*}$ strongly decreases simultaneously (Fig. 6c), the overall result is that increasing the proportion of bad years leads to less within‐season risk‐spreading (Fig. 6d–e).

Competition for space or resource depletion results in substantial dormancy even when all years are good (Fig. 6a). When late‐germinating seedlings may find the space already occupied, or fail to gather resources pre‐emptied by others, early emergence is strongly selected, which in turn can be risky when there is unpredictable variation in the start of favourable abiotic growing conditions. Dormancy is selected for as it reduces the risk that all members of a lineage germinate too early in the season; effectively it permits risky within‐season germination behaviour.

Note that early germination is also beneficial in the absence of competition and in the density‐dependent resource intake model, but in these models the benefit is only moderate; late individuals do not risk total failure. In the absence of priority effects that penalise latecomers, dormancy does not evolve if all years are good.

### Robustness and generality of results

Thus far, we assumed no reproduction in bad years and immediate death of individuals germinating before continually favourable growing conditions started. Relaxing these assumptions decreases the need for both dormancy and within‐season bet hedging, but qualitatively, the results remain unchanged; with more dormancy, less within‐season risk‐spreading is needed (Appendix S4). This pattern is robust across many parameter combinations (Appendix S5).

## Discussion

Previous theoretical work has shown that among‐year dormancy can reduce selection for other risk‐reducing mechanisms, such as iteroparity (Tuljapurkar & Wiener 2000; Koons *et al.* 2008), large seeds (Venable & Brown 1988) and dispersal (Venable & Brown 1988; but see Snyder 2006). Here, we assumed that within‐season phenology evolves independently from dormancy and demonstrated that, under this assumption, dormancy reduces the need for within‐season bet hedging. When abiotic conditions fluctuate early in the growing season, dormancy spreads germination of seeds over several years and thereby reduces the risk of failure within seasons. The ‘safety net’ of dormancy is particularly important when individuals directly compete with, and may fail to establish in the presence of, earlier germinating conspecifics. Here, priority effects cause strong selection for early phenology (analogous to migrating birds competing to arrive early, Kokko 1999), but this comes with an increased risk of harsh abiotic conditions. Should this risk materialise, the presence of a seed bank helps the lineage avoid extinction. Thus, dormancy can allow for more risky timing within a season.

We find that density dependence can make dormancy advantageous even if each growing season is favourable for reproduction. At first glance, these results resemble those of Ellner (1985a, b), in which strong density fluctuations favour dormancy, enabling a lineage to reap the benefits of germinating at low densities. Our model is, however, based on a different route to higher dormancy – instead of fluctuating population size, selection for early emergence arises via priority effects. Plants that germinate early can have a strong competitive advantage over later emerging individuals, especially at high population densities (Ross & Harper 1972; Miller *et al.* 1994; Orrock & Christopher 2010). We show priority effects to affect not only within‐season germination phenology, but also dormancy fractions.

We predict competition to increase the variance in germination timing. Both Metcalf *et al. *(2015) and Poethke *et al. *(2016) show that even in predictable environments density dependence can lead to variance in within‐season germination timing, sometimes resulting in multiple coexisting germination strategies. In these studies spreading germination within the season is a way to avoid competition; this is not the case in our study. The variance of within‐season timing evolves to manage biotic and abiotic risks, with the former favouring earlier phenology, the latter selecting for later phenology.

Our models could be extended in several directions. For example, we did not model within‐season mortality or reproducing throughout the season. Ontogenetic growth was not explicitly modelled, although differentially sized individuals can have different effects on the local environment (Ross & Harper 1972, Wang *et al.* 2010), and may respond differently to environmental challenges (Mercer *et al.* 2011; Tredennick *et al.* 2018). Taking into account individual variation (e.g. size) might, by altering the strength of competition between individuals (Rudolf 2018), permit a more detailed look at priority effects. Including within‐season mortality and continuous reproduction after reaching a certain size might even result in evolutionary branching, where multiple germination strategies coexist but reproduce at different times within the season.

Another avenue for future work is to study how variability in within‐season conditions, such as the renewal of the resource in the resource‐depletion model (e.g. another rain event which increases water availability), affects germination strategies. This could be complemented with a detailed look at germination with respect to environmental cues. Indeed, Sonoran Desert winter annuals vary in their responses to both water availability and temperature (Huang *et al.* 2016). Seeds may not only react to abiotic cues, but also to the presence of competitors (e.g. Dyer *et al.* 2000). In addition, seeds can break from their dormant state, but forgo germination and regain dormancy when germination cues are absent (Finch‐Savage & Leubner‐Metzger 2006). If cues allow some aspects of the environment to be measured or predicted, phenotypic plasticity may compete with bet hedging as a way to deal with environmental variation (Donaldson‐Matasci *et al.* 2013; Xue & Leibler 2018). Phenotypic plasticity decreases the need for bet hedging (e.g. Simons 2014), and is therefore of importance to consider in future research.

A major assumption of our model is that dormancy and within‐season timing evolve independently. This assumption allows for predictions of the combinations of germination strategies that are favoured by selection at both temporal scales. Evolution of these combinations may be difficult, however, if genetic or physiological constraints limit plants' ability to fine‐tune their responses to within‐ and among‐season patterns separately, resulting in a narrower range of patterns than documented in some studies (e.g. Torres‐Martínez *et al*. 2017). For example, Huang *et al. *(2016) showed that species that take a long time to germinate after imbibition (i.e. after seeds absorbed water) have lower among‐year germination fractions than fast germinating seeds. We expect that under such constraints seeds will evolve such that they are mainly adapted to among‐year environmental variation, and that within‐season phenology will not necessarily be optimal. However, it would be interesting to investigate if our prediction holds in the presence of priority effects.

Germination traits can also covary with other traits, such as offspring size (e.g. seed mass (Simons & Johnston 2000; Hoyle *et al.* 2015) or larval weight (Menu & Desouhant 2002). Offspring size, in turn, affects life‐history characteristics such as survival and growth, with obvious potential to influence the success of a bet hedger. To fully understand the interaction between dormancy and within‐season germination timing, therefore, requires a better understanding of traits influencing performance throughout the rest of the life cycle.

Empirical evidence on the interaction between dormancy and within‐season germination timing is limited and ambiguous (Simons & Johnston 2006; Simons 2014; Gremer *et al.* 2016; Metz *et al.* 2018). In agreement with our findings, Simons & Johnston (2006) and Simons (2014) showed a negative relation between dormancy fractions and variation in within‐season germination timing of populations of a short‐lived plant. In contrast to our findings, desert annual species that germinate early in the growing season tend to have low dormancy fractions (Gremer *et al.* 2016). A potential explanation for this discrepancy is that a combination of within‐season germination timing and high germination fractions may reflect species‐specific adaptations other than bet hedging. Among Sonoran Desert annuals, species with low dormancy and early germination are more stress tolerant, withstanding dry periods better (Kimball *et al.* 2011; Huang *et al.* 2016). A ‘good’ year for highly stress‐tolerant species may be too dry for others. Furthermore, stress‐tolerant species have lower relative growth rates (Angert *et al.* 2014), bigger seeds (Huang *et al.* 2016) and a competitive disadvantage under wet conditions (Gremer *et al.* 2013), which might all increase the need for early germination. Metz *et al. *(2018) found high germination fractions and fast germination rates for an annual grass species, despite growing in unpredictable environments. This indicates that adjusted germination behaviour via bet hedging is not a universal strategy in unpredictable environments.

Finally, although our model was developed for annual plants, the results are relevant for other organisms dealing with unpredictability. Many species use dormancy to bridge unfavourable conditions (e.g. Menu & Desouhant 2002; Furness *et al.* 2015; Tarazona *et al.* 2017), and the timing of hatching can have important fitness consequences. The dormant eggs of *Daphnia*, for example, can stay in sediments for decades. The hatching of these eggs is also spread within a season, interpretable as within‐season bet hedging to deal with uncertainty regarding the start of the season (Vanoverbeke & De Meester 2009).

Even for annual plants, life consists of a sequence of conditions operating over different time scales. Our results show that risk‐prone and risk‐averse strategies can interact with each other, with strategies at one time scale affecting the adaptive value of another. These patterns are only revealed by considering multiple time scales. We hope that our study will encourage further attention to the interactions of scales. Understanding how this drives life‐history strategies in variable environments will lend insight into the evolution of observed strategies, as well as informing responses to shifts in future conditions under climate change, which is expected to bring about further increases in environmental variability.

## Authorship

JRG and HK conceived the idea. HtB designed and analysed the models, wrote the first draft of the manuscript. All the authors contributed substantially to revisions.

## Data Availability

All data (C++ code and Maple code) is deposited to Figshare (https://doi.org/10.6084/m9.figshare.11473470 **).**
