# Supplementary material for: Optimal germination timing in unpredictable environments: the importance of dormancy for both among‐ and within‐season variation
Source: Ecol Lett. 2020 Jan 28;23(4):620–30. doi: 10.1111/ele.13461 (PMC7079161; doi:10.1111/ele.13461)
Supplement: Supplementary file 1 [file ELE-23-620-s001.docx]

**Appendix** **A: Detailed model descriptions**

In this appendix, we describe the five different models in detail. A summary of the different model assumptions can be found in table A1.

Analytical model

We assume that there are three types of years: bad years (with probability $1-\varphi$) where reproduction is not possible, late years (with probability $\varphi(1-\rho)$) that are suitable for late germinating seeds, and early years (with probability $\varphi\rho$), suitable for all germinating seeds. A seed will germinate with probability $G$, and if it does so, it will germinate early with probability $p_{e}$, and late with probability ${1- p}_{e}$. Seeds that do not germinate in a certain year have a probability of $s$ to survive in the soil. Early germinating seeds have a per capita seed production of $Y_{e}$ in early years. In case the growing conditions are poor early in the season, early seedlings do not survive. In contrast, seeds that germinate late have a per capita seed production of $Y_{\mathcal{l}},$ independent of the conditions early in the season. Since early individuals have more time to grow in case of an early year, they can gather more resources and have a higher per capita yield. We therefore assume that $Y_{e}={c_{e}Y}_{\mathcal{l}}$, where $c_{e}>1$ is the relative benefit of germinating early. Mortality of newly produced seeds before the start of the next growing season is included in the yield.

The expected contribution to the next generation of a certain lineage equals

|  | $F_{e}= G\left( {p_{e}c}_{e}Y_{\mathcal{l}}+\left( 1-p_{e} \right)Y_{\mathcal{l}} \right)+\left( 1-G \right)s$ | (A1) |
| --- | --- | --- |

in case of an early year. In a late year, the expected contribution equals

|  | $F_{\mathcal{l}}= G\left( 1-p_{e} \right)Y_{\mathcal{l}}+\left( 1-G \right)s.$ | (A2) |
| --- | --- | --- |

In bad years individuals do not reproduce and only dormant seeds contribute to the next generation according to

|  | $F_{b}= \left( 1-G \right)s.$ | (A3) |
| --- | --- | --- |

In the setting we model, the fate of a lineage is determined by its expected long-term geometric mean growth rate (Lewontin & Cohen 1969), which is given by

|  | $\varphi\rho\text{ln(}F_{e}\text{)}+ \varphi\left( 1-\rho\right)\text{ln(}F_{\mathcal{l}}\text{)}+ \left( 1-\varphi\right)\ln(F_{b})$. | (A4) |
| --- | --- | --- |

We find the optimal germination strategy by maximizing eqn A4. Note that this model simplifies to the model of Cohen (1966) when there is no variation in the start of the season and in the within-season germination timing of seeds. For example, if there are no early years ($\rho=0$) and all seeds germinate late ($p_{e}=0)$, the optimal germination fraction equals

|  | $G^{*}=\frac{\varphi Y_{\mathcal{l}} - s}{Y_{\mathcal{l}} - s}.$ | (A5) |
| --- | --- | --- |

Continuous season model

Here, we describe the continuous season model where there is a continuous variation in the day at which favorable growing conditions start. The model is an extension of the model of Poethke et al. (2016), we extend the model by including differences in the quality of each growing season.

Variation in both the quality of the season and the start of favorable growing conditions

In a specific year $t$favorable abiotic growing conditions begin on day $S_{B}(t)=\bar{S}_{B}+x_{B}(t)$. The growing season ends on day $S_{E}\left( t \right)= \bar{S}_{E}$, where $\bar{S}_{B}$ and $\bar{S}_{E}$ are the long-term average dates for onset and ending of favorable growing conditions, respectively. We assume that there is no variation in the end of the growing season $S_{E}\left( t \right)= \bar{S}_{E}$. Random values for deviations of the start of favorable growing conditions ($x_{B}(t))$ are independently drawn from the probability density function

|  | $\Phi\left( x_{B}(t) , \sigma\right)= \frac{\pi}{4\sigma}sin(\frac{\pi(x_{B}(t) +\sigma)}{2\sigma})$ for $-\sigma\leq x_{B}(t) \leq\sigma$ and 0 otherwise. | (A6) |
| --- | --- | --- |

In this equation parameter $\sigma$ determines the maximum deviation from the long-term average $\bar{S}_{B}$.

In addition to variability in the start of favorable growing conditions, the overall quality of the growing season differs among years as well. As in the analytical model above, here a growing season can either be good (with probability $\varphi$) or bad (with probability$1-\varphi$) and in bad years individuals cannot reproduce and only dormant seeds contribute to the next generation.

Germination of individuals

Each individual is characterized by three heritable traits that determine if and when an individual seed germinates in a certain season. Trait $G$ is the probability that an individual seed germinates in a certain year. We assume that this probability does not change with the age of the seed. An individual’s timing of germination within a season, $E$, is determined by its mean germination date $\bar{E}$ and its variance trait $\epsilon$ following

|  | $E= \bar{E}+y(\epsilon)$. | (A7) |
| --- | --- | --- |

In this equation $y(\epsilon)$ is drawn from the probability density function

|  | $\Phi\left( y(\epsilon),\epsilon\right)= \frac{\pi}{4\epsilon}sin(\frac{\pi(y(\epsilon)+\epsilon)}{2\epsilon})$ for $-\epsilon\leq y(\epsilon)\leq\epsilon$ and 0 otherwise. | (A8) |
| --- | --- | --- |

In the main text, we assume that individuals that germinate before the start of favorable growing conditions ($E<S_{B}(t)$) immediately die. In appendix C we relax this assumption and assume that individuals experience a daily mortality rate $\mu_{\text{early}}$ in the days before the abiotic conditions are favorable. In contrast to Poethke *et al.* (2016), we assume that there is no within-season mortality as soon as the abiotic conditions are favorable. Individual seeds that stay dormant (with probability $1-G$) have a probability $s$ to survive in the soil during the growing season. For simplicity we assume that this survival probability does not change with age.

Resource acquisition in the growing season when the year is good

For each day $\tau$ falling into the season where conditions are favorable ($S_{B}\left( t \right)\leq\tau\leq S_{E}(t)$) all currently active individuals will, in good years, collect resources at a daily rate of $c\left( \tau\right)$. We assume that individuals do not collect resources in case abiotic conditions are bad. This implies that in some years there is more time to gather resources than in others. We will consider four different models where resource acquisition does or does not depend on the strategy of other individuals.

In **the density-independent model**, the amount of resources available per day does not depend on the density of individuals in the population and equals $c(\tau)=c$, where parameter $c$ is a constant. The more days an individual is active, the more resources it can collect. The length of the season also affects season quality: years with short periods of favorable growing conditions have fewer days available for individuals to collect resources.

In the **resource-depletion model**, there is, in good years, an amount, $R,$ of resource available at the start of the season. The resource does not renew itself during the season. Each active individual collects $c(\tau)=c$ amount of resource per day, until the resource is depleted ($R=0$), after which there is no further resources available and $c\left( \tau\right)$= 0. The more days an individual is active, the more resources it can collect. Furthermore, late in the season the resource might be completely depleted, leaving no resources available for late germinating individuals, so earlier activity can be beneficial, depending on conditions. For simplicity, the total amount of resources available in a year, $R$, does not depend on the day when favorable conditions start to occur. All good growing seasons are therefore equally good in terms of total resource availability.

In the **density-dependent resource intake** **model**, the daily amount of resources an individual can gather depends on the current density of active individuals ($N\left( \tau\right)$). In this scenario there is interference competition among all individuals, where resource uptake in hindered by conspecifics. In this model we assume that individuals compete on a daily basis with each other. We assume the following relationship between daily per capita resource intake and density

|  | $c(\tau)=\frac{K\left( S_{E}\left( t \right)-S_{B}\left( t \right) \right)^{-1}}{1+aN\left( \tau\right)},$ | (A9) |
| --- | --- | --- |

where parameter $K$ determines the daily resource intake at low population density. Parameter $a$ is the competition parameter determining how strongly each competitor affects the per capita resource intake. We multiply parameter $K$ with the inverse of the length of the season such that short and long seasons are equally good in terms of total resource intake (assuming the number of competitors is equal). We choose parameters $a$ and $K$ such that on average the population produces 10.000 offspring. In appendix E we vary these parameters.

In the **competition-for-space** **model**, we assume that individuals compete for space instead of resources. Each year there are $X$ habitat patches available, that are divided upon a ‘first come first serve’ basis. An occupied patch becomes available again after an individual dies (because it germinated too early). Individuals that germinate when there are no patches available die. Due to each individual having sole ‘ownership’ of its site, daily resource intake is independent of other individuals. Daily resource intake equals $c(\tau)= c$, which is constant over the season.

For each of the four models described above the total amount of resources an individual acquires within a growing season in good years is calculated as

|  | $C\left( S_{B}\left( t \right),S_{E}\left( t \right),E \right)= \left\{ \begin{aligned} \int_{\text{max(}E, S_{B}\left( t \right))}^{S_{E}\left( t \right)} c(\tau) d\tau, &E<S_{E}\left( t \right), \\ \\ 0, &otherwise. \end{aligned} \right.$ | (A10) |
| --- | --- | --- |

Birth

At the end of the growing season, all individuals reproduce new seeds and subsequently die. The amount of seeds an individual produces, depends on its resource intake and equals $C\left( S_{B}\left( t \right),S_{E}\left( t \right),E \right)$. For simplicity we assume asexual organisms where seeds inherit their traits from their parents.

Optimal strategies

For the density-independent model we find the optimal strategy by numerically calculating the expected geometric mean fitness for all possible combinations of the traits under evolution. We use an individual based model to study the evolution of dormancy and germination times in case of the three density-dependent models (see appendix B for a detailed description of the IBM and default parameter values).

Numerical calculations were carried out in Maple (version 2016.2). The IBM was carried out in C++. The code is made available at Figshare.

**Appendix B: Individual based simulations**

We use individual based simulations to study the evolution of dormancy and within-season germination phenology in case of the three competition models in the continuous season model. We start the population with $\tilde{N}$individuals. We initialize simulations with different initial trait values to study if there are multiple evolutionary attractors. We run each simulation for 20.000 years, simulations are iterated in time steps of one day.

At the beginning of each year the start of favorable growing conditions is determined with the use of eqn A6. First, it is determined if individuals germinate or not. The seeds that do not germinate stay dormant and survive the growing season with probability $s$. We allow a maximum of 10$\tilde{N}$seeds in the soil. Seeds are removed randomly from the population when the soil population exceeds this threshold. Of the seeds that germinate in a particular year, their germination date is determines with the use of eqn A8.

Individuals germinating before the start of favorable growing conditions survive this period with probability $(1-\mu_{\text{early}})^{S_{B}\left( t \right)-E}$. In the competition-for-space model, we select $\tilde{N}$ individuals from the population of successful germinators (i.e., individuals that either germinate after the start of favorable growing conditions or that survived the harsh period before favorable growing conditions occurred), all other successful germinators are removed. We choose these $\tilde{N}$ based on their germination date, with individuals that germinate earlier occupying space that is no longer available for later germinating individuals.

During the growing season, individuals gather resources (see description appendix A), reproduce, and subsequently die. The number of offspring that an individual produce depends on the total amount of resources it has collected during the growing season (eqn A10) and is drawn from a Poisson distribution with mean $C(S_{B}\left( t \right), S_{E}\left( t \right),E)$. Mutations for each trait occur independently with probability $\upsilon$. In case a mutation occurs in one of the three traits, the inherited parental trait changes as follows: $G_{i}^{\mathrm{offspring}}=G_{i}^{\mathrm{parent}}+0.1\delta$, $\bar{E}_{i}^{\mathrm{offspring}}=\bar{E}_{i}^{\mathrm{parent}}+5\delta$, and $\epsilon_{i}^{\mathrm{offspring}}=\epsilon_{i}^{\mathrm{parent}}+2\delta$, with $\delta$ an evenly distributed random number from the interval [$-1<\delta<1]$. Changing the mutation rate $\upsilon$ or the size of the mutational steps does not change the results, but only affects the time it takes until the optimal phenotype is reached. We assume that all seeds survive the non-growing season. Parameters used in the main text are given in Table B1, model variables and evolving traits are given in Table B2. See appendix D for the robustness of our results with respect to changes in parameter values.

All simulations converged to a single optimal phenotype, independent of the initial starting conditions. We therefore plot only the mean trait values of multiple runs.

**Appendix C: Optimal fraction of dormant seeds in the presence of within-season variation**

In the main text, we studied the optimal fraction of dormant seeds as a function of within-season phenology assuming that there was no variation in the start of good growing conditions ($\rho=1$ in the analytical model and $\sigma=0$ in the density-independent continuous season model). This allowed us a clear interpretation of the results since individuals only had to deal with among-season variation. Here, we show the optimal fraction of dormant seeds in the presence of both among- and within-season variation.

When all years start early ($\rho=1$), we have for the analytical model a clearly interpretable expression for the optimal fraction of dormant seeds as a function of the fraction of late seeds (eqn. 2). Unfortunately, when there is some within-season variation ($0<\rho<1)$ the expression for the optimal dormancy fraction, ${1-G}^{*}$, as a function of the fraction of late seeds is cumbersome and not insightful. We therefore only plot the optimal dormancy fraction and do not give the analytical solution.

In the analytical model, the optimal dormancy fraction is high in case all seeds germinate early, and initially decreases with the fraction of late seeds (Fig. C1). Because of the compensatory effect of dormancy on within-season bet-hedging, the fraction of dormant seeds is high in the absence of late seeds in order to deal with the within-season variation. The higher the fraction of late seeds, the less dormancy is necessary to compensate. When the fraction of late seeds is higher than optimal (right of the dots), dormancy initially continues to decrease a bit, before it slowly increases. This non-linear response is probably a result of an interaction between lower yield and higher within-year survival with increasing fraction of late seeds.

We find a similar result in the density-independent continuous season model (Fig. C2). When either the mean emergence date, $\bar{E}$*,* or the variance,$\epsilon,$ is lower than optimal (optimal values indicated with the dots in Fig. C2), there is a high fraction of dormant seeds. The fraction of dormant seeds initially decreases with an increase in either $\bar{E}$ or $\epsilon$, and it increases again when either $\bar{E}$ or $\epsilon$ is higher than optimal. Note that the optimal dormancy fraction is much more affected by the mean emergence date $\bar{E}$ (Fig. C2a) compared to the variance in emergence date $\epsilon$ (Fig. C2b).

While we do find that within-season phenology affects the optimal dormancy fraction, this is caused by the compensatory effect of dormancy on within-season bet-hedging and not the other way around.

**Appendix D: Robustness of results with respect to mortality regimes**

In the main text we assumed that individuals emerging before favorable growing conditions started would immediately die. We furthermore assumed that bad years were fatal, such that individuals died and did not reproduce. Here, we show that changing these assumptions regarding mortality does not affect our results qualitatively. For clarity, we only show results for the joint evolution of dormancy and within-season germination time for the density-independent and competition-for-space continuous season models.

The red triangles in Figure D1 show the results in case individuals that germinate too early have a small probability to survive. We assume a daily mortality rate of 0.1 in the days before favorable growing conditions start. Individuals do not gather resources in these early days. Germinating early is less risky compared to the situation in the main text, leading to a lower optimal mean emergence date, $\bar{E}^{*}$, compared to the case where there is no survival before the start of favorable growing conditions (red dots in Fig. D1b). In the absence of competition, the variance in emergence, $\epsilon^{*}$, is lower as well (panel c in Fig. D1). Dormancy is hardly influenced by the survival rate early in the season (panel a and d in Fig. D1), and increases with the probability of encountering a bad year. Because of the compensatory effect of dormancy on within-season risk-spreading, the variance in emergence, $\epsilon^{*}$, decrease with increasing dormancy fractions for both models (panel c and f in Fig. D1). In the absence of competition, the optimal mean emergence date, $\bar{E}^{*}$, decreases as well (panel b in Fig. D1). To conclude, increasing the survival probability for individuals that germinate too early, decreases the need of within-season risk-spreading. The interaction between dormancy and within-season risk-spreading remains unaltered by this assumption.

The blue dots in Figure D1 show the results in case there is some reproduction in bad years. We assume that the daily resource intake in bad years is only 10% of that of good years (i.e., $0.1c$). This assumption leads to less dormancy in the absence of competition (panel a). As before, there is less within-season risk-spreading with increasing dormancy (panel b and c in Fig. D1). However, since there is less dormancy in case individuals can reproduce in bad years, there is more within-season risk-spreading compared to the situation where individuals did not reproduce in bad years.

When individuals compete for space, dormancy is hardly affected by the assumption that reproduction is possible in bad years (panel d in Fig. D1). Priority effects select for early emergence (panel e and f in Fig. D1), which increases the need for dormancy, even when all years are good or when bad years are not fatal.

The blue triangles in figure D1 show the results in case we assume that some reproduction is possible in bad years and individuals that germinate too early can survive. In the absence of competition, there is less need for both dormancy and within-season bet-hedging (left panels in Fig. D1). The interaction between different risk-spreading strategies remains unaffected; the more dormancy, the less within-season bet-hedging is needed.

**Appendix E: Robustness of results with respect to changes in parameter values**In this appendix, we study the robustness of our results by investigating the evolution of dormancy and within-season germination phenology for different parameter combinations in the continuous season models. We both increased and decreased standard parameter values (table B1) and show that the interaction between dormancy and within-season germination phenology remains unaltered; the more dormancy, the less within-season risk-spreading.

Figure E1 shows how changing the survival probability of dormant seeds affects the results. For clarity, we only show results for the density-independent and the competition-for-space models.

The higher the mortality rate of dormant seeds, the lower the fraction of dormancy (eqn 3a and panel a and d in Fig. E1). Therefore, there is less potential for compensation of within-season risk-spreading and the variance in emergence, $\epsilon^{*}$, increases with decreasing survival probability of dormant seeds (panel c and f in Fig. E1).

Changing the maximum numbers of individuals in the population does not affect our results qualitatively (Fig. E2). In the competition-for-space model, we calculate the optimal strategy in case of $X= 0.5\tilde{N}$, $\tilde{N}$, and $\tilde{2N}.$ For the resource-depletion model, we assume at the beginning of each growing season $0.5R$, $R$, and $2R$ amount of resources. For the density-dependent resource intake model, we either assume values of $K$ of 3.5, 6, and 11 while keeping parameter $\alpha$ constant, or we assume values of parameter $\alpha$ of 0.00025, 0.0005, and 0.001, while keeping parameter $K$ constant. Changing parameters $K$ or $\alpha$ changes the resource intake of individuals and thereby affects the population size. The higher parameter $K$, the larger the population can grow. Vice versa, high values for the competition parameter $\alpha$ decreases the maximum size of the population. While changing the population size does not change the results in the competition-for-space and the resource-depletion models (panel a-f in Fig. E2), it does have a small effect on within-season germination phenology in the density-dependent resource intake model in case parameter $K$ is changed (panel g-i in Fig. E2), but not when competition parameter $\alpha$ is changed (panels j-l in Fig. E2). The higher parameter $K$, the lower the mean emergence date, $\bar{E}^{*}$, and the higher the variance in emergence, $\epsilon^{*}$. Since daily resource intake is close to $K$ when the population size is small (eqn A9), germinating before everyone else is extremely beneficial when parameter $K$ is high. It is therefore advantageous to produce offspring that vary a lot in their within-season germination timing, to ensure that some germinate as soon as favorable growing conditions start.

Figure E3 shows the optimal trait values for two different values of $c$, the daily resource intake. We assumed a default daily resource intake of $c=0.05$, which results in on average 5 offspring if an individual grows for 100 days. Lower values of parameter $c$ leads to the extinction of the population (results not shown), higher values of parameter $c$ leads to less dormancy (Fig. E3), which is also predicted by the analytical model (eqn. 3a). The interaction between dormancy and within-season germination phenology remains unaltered; the more dormancy, the less within-season risk-spreading. In the absence of competition, the effect of dormancy on within-season germination timing is limited when daily resource intake $c$ is high (panels b and c in Fig. E3). In both competition models, a higher intake rate leads to more spread and a lower mean emergence date.

**Table** **A1:** Overview of the different models and their assumptions

|  | **Analytical model** | **Continuous season model** | | | |
| --- | --- | --- | --- | --- | --- |
|  |  | ***Density-independent*** | ***Competition-for-space*** | ***Resource-depletion*** | ***Density-dependent resource intake*** |
| **Quality of growing seasons** | Either good or bad | Either good or bad | Either good or bad | Either good or bad | Either good or bad |
| **Start of good growing conditions** | Either early or late | Continuous distribution between $\bar{S}_{B}-\sigma$ and $\bar{S}_{B}+\sigma$ | Continuous distribution between $\bar{S}_{B}-\sigma$ and $\bar{S}_{B}+\sigma$ | Continuous distribution between $\bar{S}_{B}-\sigma$ and $\bar{S}_{B}+\sigma$ | Continuous distribution between $\bar{S}_{B}-\sigma$ and $\bar{S}_{B}+\sigma$ |
| **Mortality risk for early individuals** | Early individuals always die when they germinate in a late year | Probability of $\mu_{\text{early}}$ to die per day before good growing conditions start.  $\mu_{\text{early}}=1$ in main text and 0.1 in App D | Probability of $\mu_{\text{early}}$to die per day before good growing conditions start.  $\mu_{\text{early}}=1$ in main text and 0.1 in App D | Probability of $\mu_{\text{early}}$to die per day before good growing conditions start.  $\mu_{\text{early}}$ = 1 | Probability of $\mu_{\text{early}}$to die per day before good growing conditions start.  $\mu_{\text{early}}=1$ |
| **Within-season germination time** | A fraction $p_{\text{e}}$of seeds germinates early, the rest late. | Continuous distribution between $E-\epsilon$ and $E+\epsilon$ | Continuous distribution between $\bar{E}-\epsilon$ and $\bar{E}+\epsilon$ | Continuous distribution between $\bar{E}-\epsilon$ and $\bar{E}+\epsilon$ | Continuous distribution between $\bar{E}-\epsilon$ and $\bar{E}+\epsilon$ |
| **Space availability** | Infinite | Infinite | $X$, based on first-come-first serve basis | Infinite | Infinite |
| **Resource availability** | Infinite | Infinite | Infinite | $R$ | Infinite |
| **Resource intake in good years** | $Y_{\mathcal{l}}$ for late seeds  $Y_{e}$ for early seeds in early years | $c$ for each day an individual is active | $c$ for each day an individual is active | $c$ as long as there is resource available | $\frac{K\left( S_{E}\left( t \right)-S_{B}\left( t \right) \right)^{-1}}{1+aN\left( \tau\right)}$ for each day an individual is active |
| **Resource intake in bad years** | 0 | 0 in main text  0.1$c$ in appendix D | 0 in main text  0.1$c$ in appendix D | 0 | 0 |
| **How is the optimal strategy calculated?** | Analytically | Numerically | Individual based simulations | Individual based simulations | Individual based simulations |

**Table B1**: Model parameters

| Parameter | Description | Default value |
| --- | --- | --- |
| $\bar{S}_{B}$ | Mean start of favorable growing conditions | 100 |
| $\bar{S}_{E}$ | Mean end of the season | 200 |
| $\bar{S}_{E}-\bar{S}_{B}$ | Average length of the season (days) | 100 |
| $\sigma$ | Maximum deviation of the start of favorable growing conditions (days) | 0 - 60 |
| $\varphi$ | Probability of encountering a good year | Between 0.5 and 1 |
| $c$ | Amount of resources an individual can acquire in one day in a good year (g day^-1^) | 0.05 |
| $s$ | Probability of dormant seeds to survive the growing season | 0.9 |
| $\mu_{\text{early}}$ | Daily mortality rate before the start of favorable growing conditions | 1 |
| $\tilde{N}$ | Initial population size | 10.000 |
| $X$ | Number of patches available in the competition-for-space model | $\tilde{N}$ (=10.000) |
| $R$ | Amount of resources at the beginning of the growing season in the resource-depletion model (g) | $\tilde{N}$ (=10.000) |
| $K$ | Maximum total resource intake at low densities in the density-dependent resource intake model (g) | $6$ |
| $a$ | Competition coefficient in the density-dependent resource intake model (individual^-1^) | 0.0005 |
| $\upsilon$ | Mutation probability in the individual based models | 0.001 |

**Table B2:** Model variables and evolving traits

| **Variable** | **Description** |
| --- | --- |
| $S_{\text{B}}\left( t \right)=\bar{S}_{\text{B}}+x_{\text{B}}(t)$ | Start of continuously favorable growing conditions |
| $x_{\text{B}}\left( t \right)$ | Deviation of the mean starting date (days). This value is each year drawn from the probability distribution described with equation A6. |
| $\tau$ | Day in the growing season |
| $\varphi$ | Probability of encountering a year with favorable growing conditions |
| $G$ | Germination probability of an individual in a certain year |
| $\bar{E}$ | Mean emergence date of an individual |
| $\epsilon$ | Variance in emergence date of an individual |
| $E=\bar{E}+y(\epsilon)$ | Day of emergence of an individual |
| $y(\epsilon)$ | Deviation of the mean emergence date (days). This value is for each individual drawn from the probability distribution described with equation A8. |
